# Immunotherapy of osteosarcoma based on immune microenvironment modulation

**DOI:** 10.3389/fimmu.2024.1498060

**Published:** 2025-01-23

**Authors:** Heping Lian, Jiakui Zhang, Shuna Hou, Shuang Ma, Jiachen Yu, Wei Zhao, Duoyi Zhao, Zhiyu Zhang

**Affiliations:** ^1^ Department of Orthopedics, The Fourth Affiliated Hospital of China Medical University, Shenyang, China; ^2^ Bone and Soft Tissue Tumours Research Centre of Yunnan Province, Department of Orthopaedics, The Third Affiliated Hospital of Kunming Medical University (Yunnan Cancer Hospital, Yunnan Cancer Center), Kunming, China; ^3^ Department of Surgical Oncology, The Fourth Affiliated Hospital of China Medical University, Shenyang, China; ^4^ Department of General Surgery, The Fourth Affiliated Hospital of China Medical University, Shenyang, China; ^5^ Nursing Department, The Fourth Affiliated Hospital of China Medical University, Shenyang, China

**Keywords:** immunotherapy, immune microenvironment, biomaterials, targeted therapy, osteosarcoma

## Abstract

Osteosarcoma is a highly malignant tumor with unsatisfactory therapeutic outcomes achieved by chemotherapy, radiotherapy, and surgery. As an emerging oncological treatment, immunotherapy has shown potential in the clinical management of many tumors but has a poor response rate in osteosarcoma. The immunosuppressive microenvironment in osteosarcoma is the main reason for the ineffectiveness of immunotherapy, in which the low immune response rate of immune effector cells and the high activation of immunosuppressive cells contribute to this outcome. Therefore, modulating the function of the immune microenvironment in osteosarcoma is expected to remodel the immunosuppressive microenvironment of osteosarcoma and enhance the efficacy of immunotherapy. This article reviews the role of immune cells in the progression of osteosarcoma, describes the corresponding regulatory tools for the characteristics of different cells to enhance the efficacy of osteosarcoma immunotherapy, and concludes the prospects and future challenges of osteosarcoma immunotherapy.

## 1 Introduction

As a highly malignant tumor, osteosarcoma predominantly affects adolescents and the aged (1–3). Patients with untreated osteosarcoma usually have a poor prognosis, with a low 5-year survival rate, and are typically accompanied by distant metastases (4–6). According to the National Comprehensive Cancer Network (NCCN) guideline, the main treatments for bone tumors include surgery, radiotherapy, and chemotherapy. Still, the treatment results are unsatisfactory, and often accompanied by recurrence (7). Surgical treatment is usually more effective in patients who have not developed metastases, however, it is less effective in patients who have developed metastases. The addition of neoadjuvant chemotherapy has increased the five-year survival rate of patients to 50-60%. However, the ensuing problems of chemotherapy drug resistance and drug toxicity remain challenging (8, 9).

Immunotherapy, despite some successes, remains helpless against osteosarcoma. Cytokine therapy is usually associated with severe systemic side effects due to tumor suppression by high-dose administration (10). Immune checkpoint blockade therapies appear helpless in the face of tumors that lack immune infiltration (11). Chimeric Antigen Receptor T-Cell (CAR-T) therapies have not achieved excellent results in clinical trials for a wide range of solid tumors (12). Researchers are working to regulate the tumor immune microenvironment, enhance immune cell infiltration and anti-tumor effects, and improve the efficacy of tumor immunotherapy by searching for new targets for immunotherapy, building a novel drug delivery platform, and combining multiple immunotherapies (13).

Since the advent of immunotherapy, it has achieved satisfactory results in hematologic tumors, melanoma, and other tumors (14). The use of immunotherapy in osteosarcoma has been inhibited by the strongly immunosuppressive microenvironment of osteosarcoma as well as by the lesser infiltration of immune cells. Cytokine therapy was one of the first therapies used in tumor immunotherapy. Interferon-alpha was approved for treating leukemia in 1986, followed by IL-2 for treating metastatic renal cell carcinoma and advanced melanoma (15). Immune checkpoint blockade (ICB) therapy has also been approved by the U.S. Food and Drug Administration (FDA) for clinical use (16). FDA-approved treatments include Ipilimumab for CTLA-4, pembrolizumab and nivolumab for PD-1, and atezolizumab, durvalumab, and avelumab for PD-L1 (17–19). CAR-T therapy has also been used for hematologic tumors and has progressed in some solid tumors. Since 2017, CAR-T therapies have been approved for hematologic tumors such as acute lymphoblastic leukemia, B-cell lymphoma, and relapsed or refractory mantle cell lymphoma. The approval of Iovance’s non-transgenic tumor-infiltrating lymphocyte (TIL) therapy lifileucel (Amtagvi) in February of this year, making it the first FDA-approved cellular therapy product for solid tumors (metastatic melanoma), may signal the future of CAR-T’s boon to solid tumor patients (20–23).

**Figure 1:**
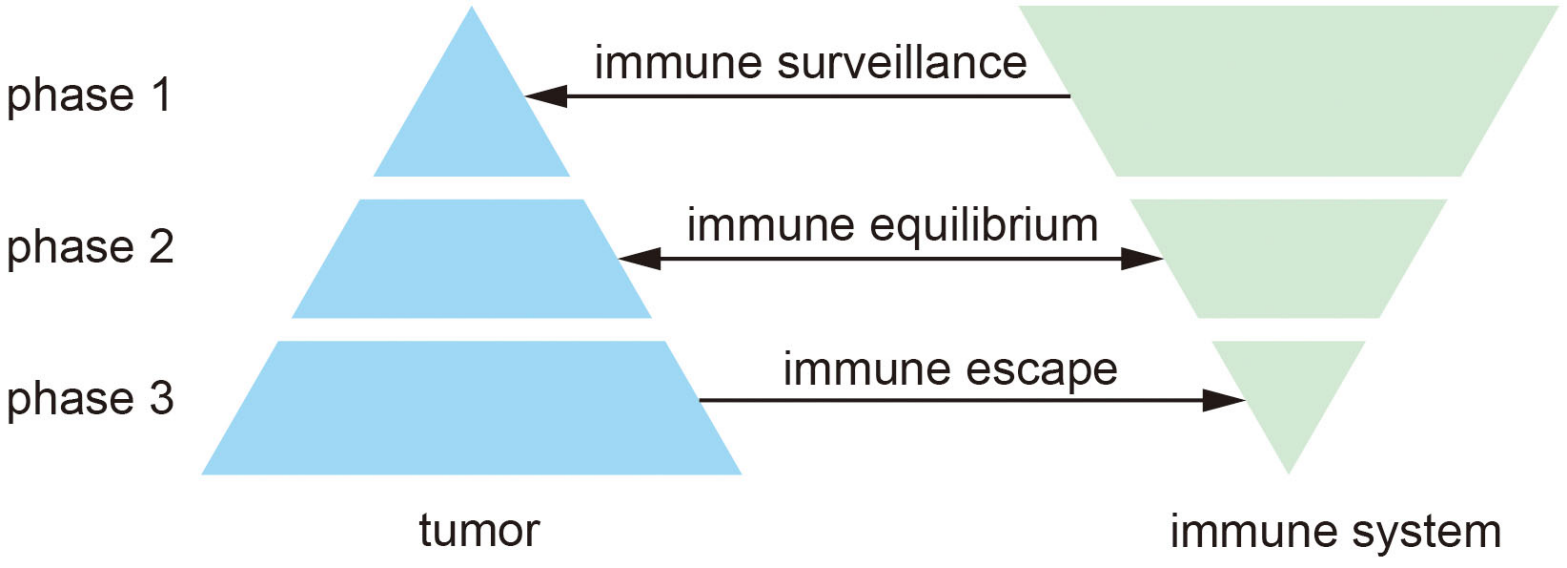
Three phases of cancer immunity editing.

Tumor immunotherapy is closely associated with the tumor immune microenvironment (TIME), which encompasses immune cells, various cytokines, and chemokines, tumor-derived exosomes, among other components, and plays a crucial role in tumor progression (24). TIME is critical to the prognosis of tumor immunotherapy and has been studied extensively (25). According to the theory of cancer immunoediting, the interaction between the tumor and the immune system can be divided into three stages (**Figure 1**): immune surveillance, immune equilibrium, and immune escape (26). In the resistant surveillance stage, the leading role of the tumor immune microenvironment is to remove tumor cells. Still, when tumor cells escape from surveillance and start to mutate, they enter the resistant homeostasis stage. In this stage, the immune system can no longer altogether remove tumor cells, while tumor cells cannot increase rapidly, and they form a dynamic balance. After continuous mutation, tumor cells can eventually evade the surveillance of the immune system, start to grow, and finally, develop into a tumor and enter the immune escape stage (27, 28). In the early stages of tumor development, TIME usually inhibits tumor progression, and as tumors gradually evade immune surveillance, TIME promotes tumor progression in the presence of the tumor.

**Figure 2:**
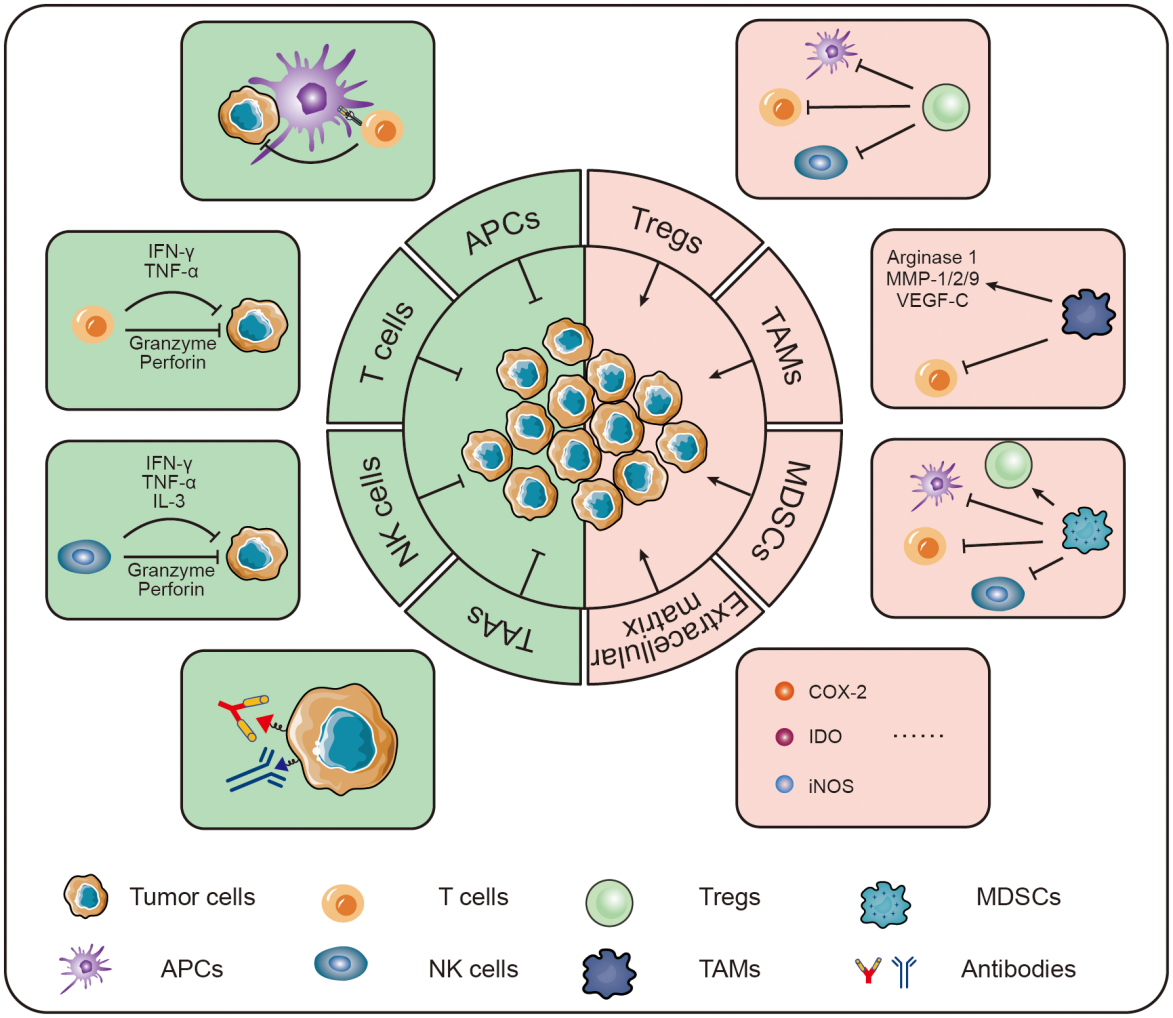
Strategies for immunotherapy of osteosarcoma.

This article highlights an overview of some of the new immunotherapeutic strategies that have emerged in recent years that ultimately lead to immunotherapy of bone tumors by enhancing immunity and reversing the immune microenvironment (**Figure 2**).

## 2 Enhance the response in the tumor immune microenvironment

In the tumor immune microenvironment, many immune cells and cytokines play anti-tumor roles, which inhibit tumor growth by attacking tumor cells or secreting cytokines (29, 30). In the immunotherapy of bone tumors, tumor progression can be inhibited by enhancing the responsive regulation of TIME by immune effector cells (31, 32). People have enhanced the immune response in the immune microenvironment of bone tumors by improving the response of antigen-presenting cells, enhancing the tumor-killing activity of T cells, enhancing the tumor-killing activity of NK cells, enhancing the anti-tumor capacity of M1-type macrophages, and enhancing systemic immunity.

### 2.1 Enhance antigen presentation by antigen-presenting cells

Antigen-presenting cells (APCs), an essential component of the immune system, can take up specific antigens, process them, and express them as antigenic peptide/MHC molecular complexes on the cell surface, where they are recognized by T cells in tumor-draining lymph nodes and activated to produce an immune response (33). In the immune microenvironment of bone tumors, APCs are mainly dendritic cells (DCs) (34). The conserved status of DCs in solid tumor infiltration was reviewed by Gerhard et al. They activate initial T cells and turn on a specific immune response after recognizing the tumor-associated antigen (TAA) (35).

In a preclinical study in 2006, Joyama et al. were the first to report that DC immunotherapy was effective against pulmonary metastasis of osteosarcoma in mice (36). They used tumor lysates to stimulate DCs, which were inoculated on LM8 tumor-bearing mice and effectively inhibited the progression of osteosarcoma and lung metastasis in mice, a result that has attracted interest. Miwa et al. reported a phase I/II clinical trial using DC and autologous tumor lysates for bone and soft tissue sarcoma conducted from 2008-2014, in which patients’ peripheral blood mononuclear cells (PBMCs) were extracted and induced into DCs and treated with autologous tumor lysates, TNF-α, and OK-432 (37, 38). The DCs were then injected into the patients to observe the adverse and treatment effects. In all 37 patients, no treatment-related adverse reactions were found. Patients had significantly elevated serum IFN-γ and IL-12 levels, suggesting that DC immunotherapy activated the immune response of the patients. However, most of these patients had to undergo other treatments because of tumor progression. This suggests that we cannot satisfy the anti-tumor needs by enhancing the strength of the DC-activated immune system alone. Therefore, DC-based immunotherapy needs to be used in combination with other therapies.

**Figure 3:**
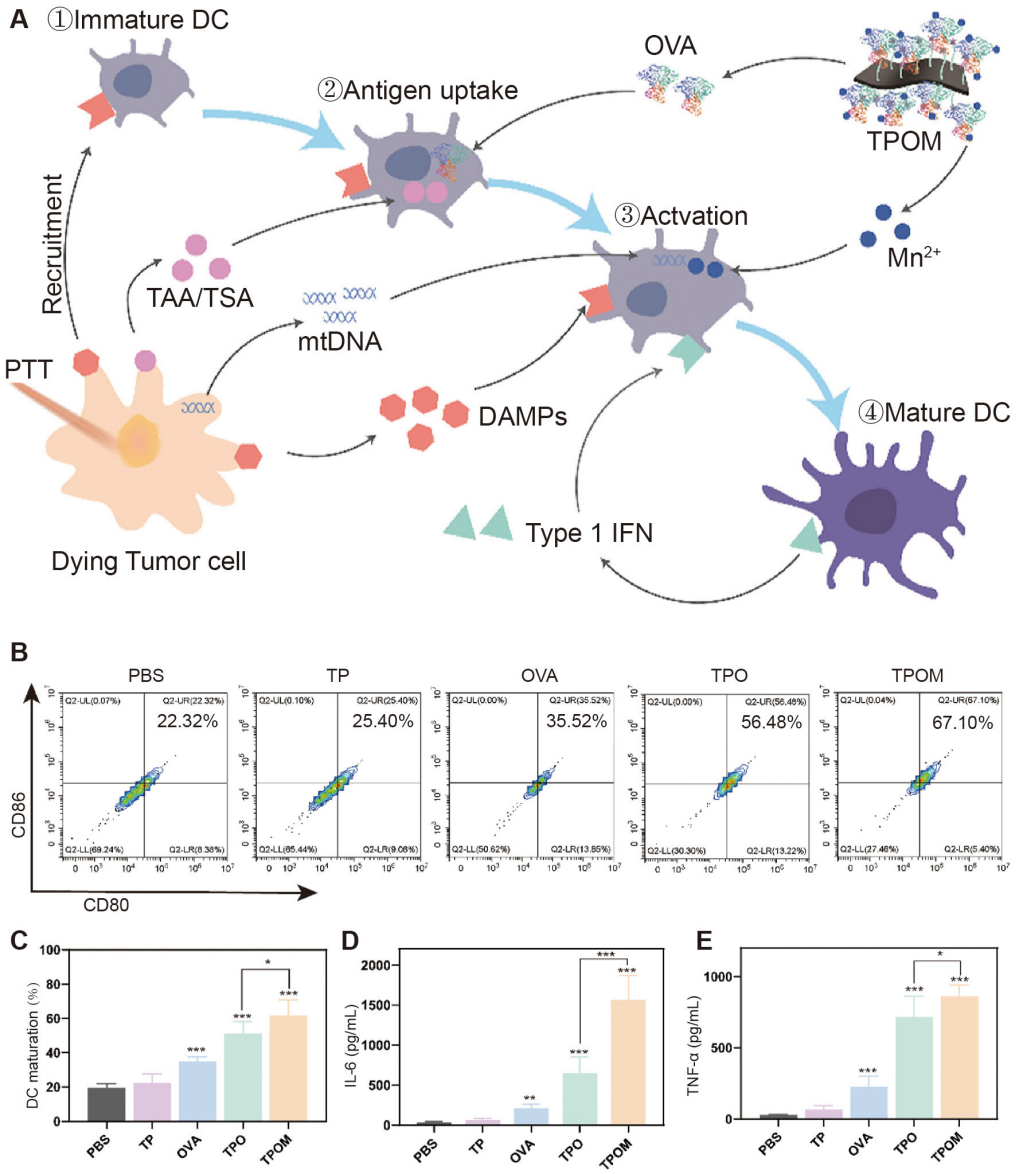
TPOM nanoparticles induced mt-DNA release and DC maturation in tumor cells after irradiation *in vitro*. **(A)** Schematic illustration of TPOM mediated immunotherapy based on PTT throughout the whole process of DCs mature. **(B, C)** Flow cytometric analysis and quantification of mature DCs (CD86+/CD80+, gated on CD11c+ cells) at 24 h after various treatments including PBS, TP, OVA, TPO, and TPOM. **(D, E)** IL-6 and TNF-a secreted in the culture medium by BMDCs (39).* *P*< 0.05, ** *P*< 0.01, *** *P*< 0.001.

Liu et al. enhanced effective DC cell presentation by photothermally triggered immunogenic monotherapy (**Figure 3**) (39). Combining titanium carbide with manganese ion-containing ovalbumin (OVA) to form a nano platform, the release of mt-DNA and Mn2+ under near-infrared laser irradiation synergistically activated the immune system and enhancing the antigen presentation of DCs, thereby enhancing the tumor-killing ability of cytotoxic T lymphocytes (CTL). This study significantly activated natural and passive immunity and effectively inhibited primary and distant tumor progression in a subcutaneous model of osteosarcoma in LM8 mice. The glucocorticoid-induced tumor necrosis factor receptor (GITR) family-related protein is expressed at high levels on Tregs. Kawano et al. investigated the anti-tumor effects of DCs combined with anti-GITR antibodies on osteosarcoma (40). In a subcutaneous model of osteosarcoma in LM8 mice, treatment with anti-GITR antibodies or DCs alone showed little difference compared with the control group. Still, when both were used in combination for treatment, tumors were significantly suppressed. Meanwhile, TGF-β, IL-10, and IL-6 expression significantly decreased, and more CD8+ T cells were recruited to the tumor area. These studies of enhancing DCs in combination with other immunotherapies suggest that how the antigen-presenting ability and T-cell recruitment capacity of DCs can be used as a critical immunotherapeutic adjunct should be further investigated and developed.

### 2.2 Enhance tumor killing by T cells

T cells play a direct tumor-killing role in the immune system; however, when tumors progress to the immune escape stage, the anti-tumor capacity of T cells is suppressed (41). In addition, T-cell infiltration in bone tumor tissues is undesirable, and less T-cell infiltration is often associated with a poorer prognosis (42). Therefore, enhancing the tumor-killing ability of T cells is a current focus of bone tumor immunotherapy.

CD8+ cells play an important role in tumor clearance as an essential part of tumor-specific immunity (43). Unfortunately, however, CD8+ cells infiltrate significantly less in bone tumors than in other solid tumors (44). Casanova et al. noted that the rate of CD8+ infiltration is an important influence on the survival of patients with bone tumors (45). This was also demonstrated in another clinical analysis of sarcomas, where the killing of tumor cells by CD8+ T cells was often blocked by immune checkpoints on the tumor, such as PD-L1 and CTLA-4, and in recent years there has been a flurry of activity regarding ICB therapies (46, 47).

**Figure 4:**
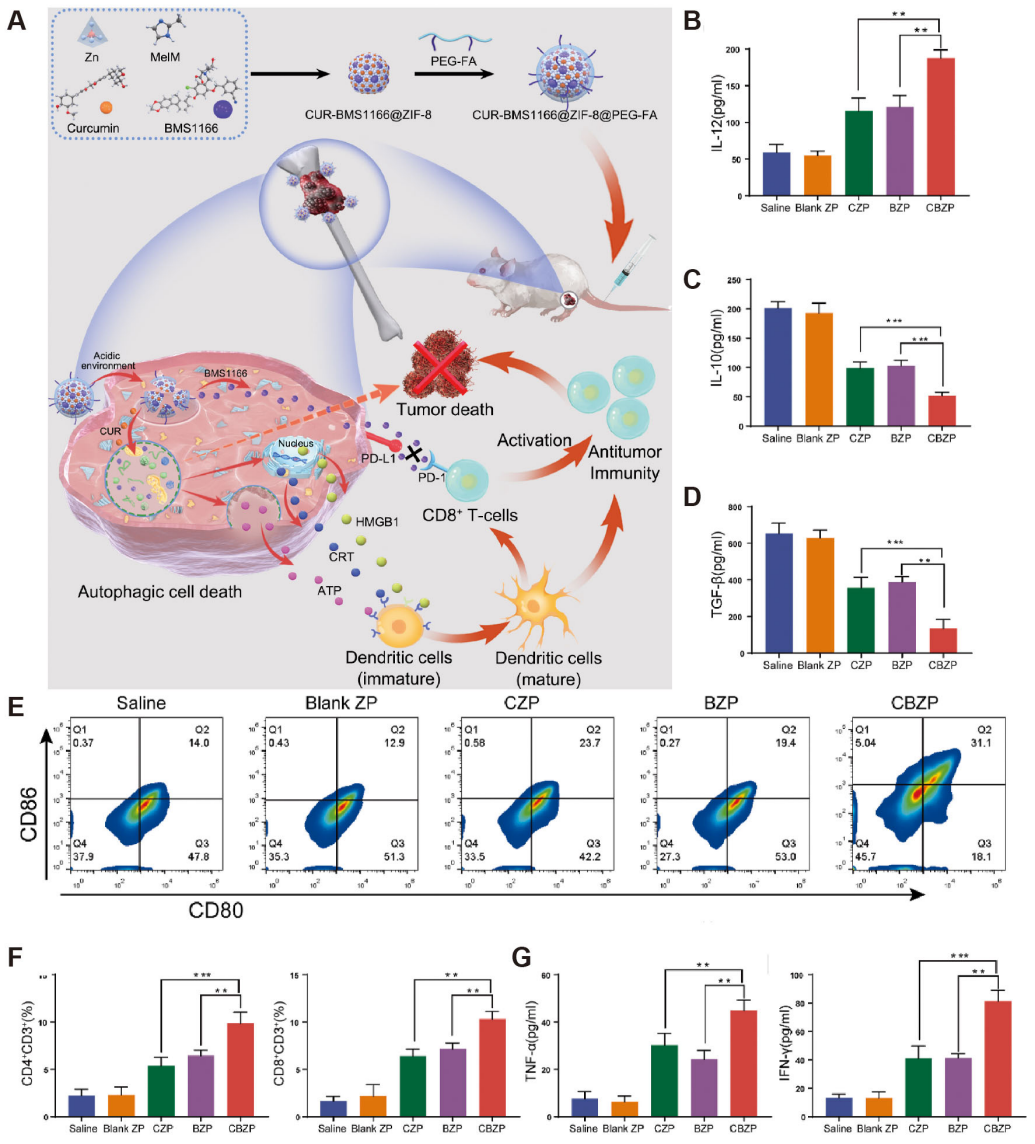
CBZP remodels the immune microenvironment of OS. **(A)** Flow cytometry shows the percentage of matured DCs from the total number of DCs in the draining lymph nodes. **(B–D, G)** Cytokine levels in serum samples from mice after different treatments. **(E)** The percentage of matured DCs from the total number of DCs in the draining lymph nodes by flow cytometry. **(F)** Quantitative evaluation of intratumoral infiltration of cytotoxic CD8+ T cells and CD4+ T cells via flow cytometry. Quantitative evaluation of intratumoral infiltration of cytotoxic CD8+ T cells and CD4+ T cells via flow cytometry (51).

Sundara et al. found high PD-L1 expression in patients with metastatic osteosarcoma, so anti-PD-1/PD-L1 therapy in this group of patients may result in a better prognosis (48). In a clinical trial, 60 patients with osteosarcoma presenting with metastases received TIL infusion combined with anti-PD-L1 therapy, and only 2 (3.33%) patients experienced grade 3 or 4 treatment-related adverse effects. 22 of 60 (36.6%) patients experienced tumor regression, and the results showed that TIL infusion combined with anti-PD-L1 therapy prolonged the survival of chemotherapy-resistant metastatic in patients with osteosarcoma (49). He et al. explored the immune response generated by combining L-arginine and anti-PD-L1 antibodies for treating osteosarcoma in mice (50). They found that L-type arginine significantly increased the number of CD8+ T cells, serum IFN-γ, granzyme B, and perforin in the spleen, while anti-PD-L1 antibody effectively prevented T cell depletion, and the two synergistically prolonged the survival of K7M2-bearing mice. Ge et al. used metal-organic nanoparticles to modulate osteosarcoma autophagy and enhance anti-PD-1/PD-L1 immunotherapy (**Figure 4**) (51). After enhanced autophagy and immune checkpoint blockade treatment, tumor tissue flow patterns of K7M2 tumor-bearing mice showed that tumor cell autophagy was significantly enhanced, the number of CD8+ T cells and DCs were significantly increased, and tumor growth was significantly inhibited.

In addition to immune checkpoint blockade therapy, other approaches to enhance CD8+ T cells have been explored. Sand et al. found by gene sequencing that CCL21 expression in Ewing sarcoma samples was negatively correlated with metastasis, primarily by non-tumor-infiltrating immune cells in the samples, suggesting an immunotherapeutic target for Ewing sarcoma (52). Yahiro et al. explored the activation of the Toll-like receptor-4 pathway to stimulate CD8+ T cytotoxic lymphocytes to inhibit osteosarcoma progression (53). The use of lipopolysaccharide (LPS) in the LM8 cell line to activate TLR-4 resulted in the discovery of more CD8+ T cells in the tumor metastases and the suppression of tumor volume in the primary foci.

In the last decade, the ability of T cells to recognize and kill tumors has been enhanced by chimeric antigen receptor (CAR) technology. CAR-T cell therapy has yielded excellent results in hematologic tumors and some solid tumors. Proven CAR-T cell therapeutic targets for bone tumors include HER2, GD2, and B7-H3, and many efforts have been made to treat bone tumors using CAR-T cell technology.

Talbot et al. constructed a new model of *in situ*, spontaneously metastatic osteosarcoma using LM7 cells expressing firefly luciferase (LM7.ffLuc) for real-time imaging of tumor metastasis (54). In this model, they investigated the anti-tumor activity of B7-H3-CAR-T cell therapy and detected tumor metastasis. In cellular assays, the B7-H3-CAR-T cell therapy group secreted significantly more IFN-γ and IL-2 than the control group. In animal experiments, the therapy demonstrated dose-dependent anti-tumor effects, with no tumor progression and no detectable tumor cells in amputated bone sections in the applied medium-high dose and high dose treatment groups, demonstrating the potent anti-tumor ability of CAR-T cells. Majzner et al. explored the anti-tumor effects of B7-H3-CAR-T cell therapy in osteosarcoma, Ewing sarcoma, and medulloblastoma (55). Charan et al. inadvertently discovered that hepatocyte growth factor (HGF) enhances GD2 expression on the surface of Ewing sarcoma cells and achieves unexpected anti-tumor effects when combined with GD2-CAR-T cells for the treatment of Ewing sarcoma (56). Similarly, Kailayangiri et al. used an EZH2 inhibitor to enhance GD2 expression on the surface of Ewing sarcoma cells and significantly inhibited tumor progression when combined with GD2-CAR-T cells for the treatment of Ewing sarcoma (57).

αβ T cells and γδ T cells are two distinct subsets of T lymphocytes that play key roles in the body’s immune response. αβ T cells, which are the most common type of T cells, express either CD4 or CD8 co-receptors and rely on recognition of antigens presented by major histocompatibility complex (MHC) molecules. In contrast, γδ T cells are a unique subset of T cells that lack CD4 and CD8 co-receptors and do not require antigen presentation through MHC molecules. Although γδ T cells are typically less abundant in tumors compared to CD4+ and CD8+ T cells, they possess a more potent tumor-killing ability. These cells can directly recognize and respond to a wide range of tumor-associated antigens without MHC restriction, and they do not require helper cells for activation. This makes γδ T cells an attractive target for immunotherapy, particularly in overcoming some of the limitations faced by traditional αβ T cell-based therapies.

Sun et al. found that zoledronate enhanced the killing of chondrosarcoma by γδ T cells in pericyte therapy (58). Caroline M Hull et al. substituted the caspase 1 cleavage site within pro-IL18 with that preferred by granzyme B, yielding GzB-IL18. They demonstrated that GzB-IL18 enhances the efficacy of αβ and γδ CAR T cell immunotherapy in a tumor-dependent manner and that GzB-IL18 provides a highly effective armoring strategy for γδ CAR T cells. GzB-IL18 promotes anti-tumor activity and myeloid cell reGzB-IL18 promotes anti-tumor activity and myeloid reprogramming without causing CAR-T cell-mediated cytokine release syndrome (59).

### 2.3 Enhance tumor killing by natural killer cells

NK cells are generally considered to be derived directly from the bone marrow, and their developmental maturation depends on the bone marrow microenvironment (60). MHC does not limit the killing activity of NK cells and does not depend on antibodies. Therefore it is called natural killing activity (61). The cytoplasm of NK cells is abundant and contains large asplenophilic granules, and the content of granules is positively correlated with the killing activity of NK cells (62). The anti-tumor effect of NK cells is very rapid, and the killing effect is seen *in vivo* in about 4 hours (63). NK cells can be activated by IL-15 and secrete IFN-γ to regulate the tumor immune microenvironment (64).

**Figure 5:**
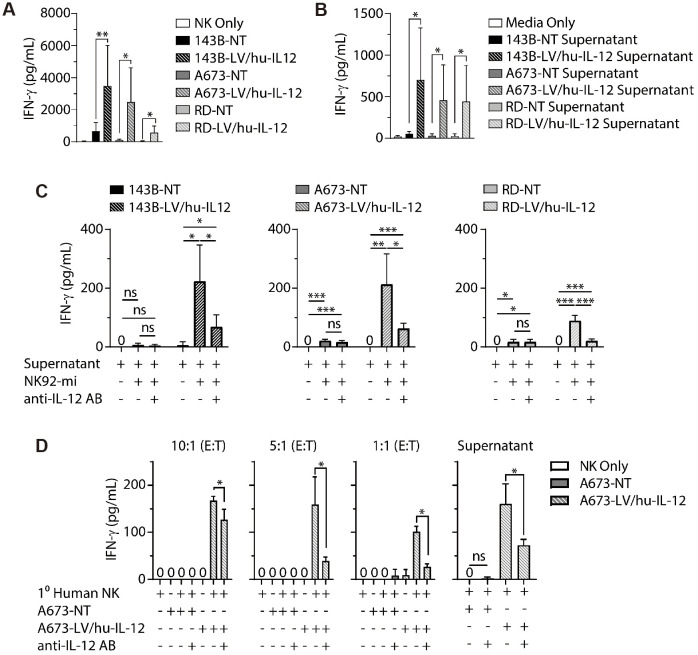
LV/hu−IL−12 transduction induces NK cell−mediated IFN−g production. **(A)** Non-transduced (NT) and LV/hu-IL-12 transduced human sarcoma lines for osteosarcoma (143B), Ewing sarcoma (A673), and rhabdomyosarcoma (RD) were plated in ultralow adherent 96-well plates. After 48 h of growth, media or NK-92mi at a 10:1 ratio were added. ELISA assessed supernatants for IFN-g. **(B)** Non-transduced and LV/hu-IL-12 transduced human sarcoma lines were plated. 4 h later, conditioned supernatant was collected and 100 µL applied to NK-92mi cells. After an additional 4 h supernatant was collected, and IFN-g was measured by ELISA. **(C)** NK-92mi cells were incubated in the presence or absence of anti-IL-12 antibody and 100 µL of conditioned supernatant. After 4 h supernatant was collected, and IFN-g was measured by ELISA. **(D)** Primary human NK cells were plated with nontransduced (NT) and LV/hu-IL12 transduced human Ewing sarcoma (A673) at the noted E:T ratios or conditioned supernatant (100 µL) in the presence or absence of anti-IL-12 antibody. After 6 h supernatant was collected, and IFN-g was measured by ELISA (67). * *P*< 0.05, ** *P*< 0.01, *** *P*< 0.001.

Fernández et al. determined that NK cells recognize osteosarcoma cells and cause osteosarcoma cell lysis through the binding between the NKG2D receptor and NKG2D ligand (65). They also reported that spironolactone appeared to enhance the sensitivity of this process. At the same time, Buddingh et al. used IL-15 activation of NK cells to enhance the ability of NK cells to kill osteosarcoma cells (66). IL-15 activation significantly enhanced cytotoxicity, and osteosarcoma cells were susceptible to such NK cells activated by IL-15. Successful inhibition by IL-15-activated NK cells was still observed in osteosarcoma cells from patients resistant to chemotherapeutic agents, while Rademacher et al. reported that IL-12 expression in sarcoma promoted immune regulation by NK cells (**Figure 5**) (67). In osteosarcoma, Ewing sarcoma, and rhabdomyosarcoma cell lines, increased IL-12 expression levels by lentiviral transduction successfully induced elevated levels of IFN-γ release from NK cells *in vitro* without systemic toxicity due to IL-12 injection, which provides a new idea for NK cell-based tumor immunotherapy. Jamitzky et al., on the other hand, found that inhibition of insulin-like growth factor-1 receptor (IGF-1R) significantly promoted the proliferation of human NK cells and could be used to combat Ewing sarcoma (68).

### 2.4 Enhance tumor killing by activating the systemic immune system

As mentioned above, there are a large number of immune cells and immunomodulatory factors in the tumor immune microenvironment that play an immunomodulatory role, and these factors are not independent of each other but affect each other (69). People have also tried to suppress tumors by enhancing the body’s overall immunity. The first idea is to activate immunity through vaccination to enhance the immune system to recognize and kill tumors (70). Tumor autoantigens have become the leading research direction of tumor vaccines, and the injection of artificially treated tumor antigens into the body can effectively activate the anti-tumor activity of the immune system. Direct targeting of specific tumor antigens is also a new immunotherapeutic modality that enhances the killing effect on tumors by mobilizing the body’s immune cells to the tumor site (71). Tumor-associated antigens such as GD2, HER2, and B7-H3 are highly expressed in bone tumors, so this is a promising research direction for immunotherapy. In current studies, this therapy usually plays an adjuvant role and is combined with other immunotherapies.

Flesner et al. successfully inhibited tumor progression and prolonged survival in dogs using autologous cancer cell inoculation, passaged T-cell therapy, and injected interleukin-2 in a canine osteosarcoma model (72). In the phase II clinical trial that included 20 patients with bone and soft tissue sarcoma, patients of different subtypes were vaccinated with a personalized peptide vaccine, and no adverse effects associated with vaccination were observed in the patients. In this study, lung metastases were reduced in 20 patients with a median survival of 9.6 months, suggesting the potential of this therapy for widespread use in patients with advanced refractory sarcomas. Li et al. combined heat shock protein/peptide immunotherapy with immune checkpoint inhibition therapy (73). Heat shock proteins can act as carriers to carry tumor antigenic peptides and can be used as a tumor vaccine. In a mouse osteosarcoma model, this vaccine was combined with anti-PD-L1 immune checkpoint inhibition therapy to inhibit tumor growth and metastasis. At the same time, Evans et al. reported a YLNPSVDSV peptide that can be used as a specific antigen for immunotherapy in Ewing sarcoma (74).

**Figure 6:**
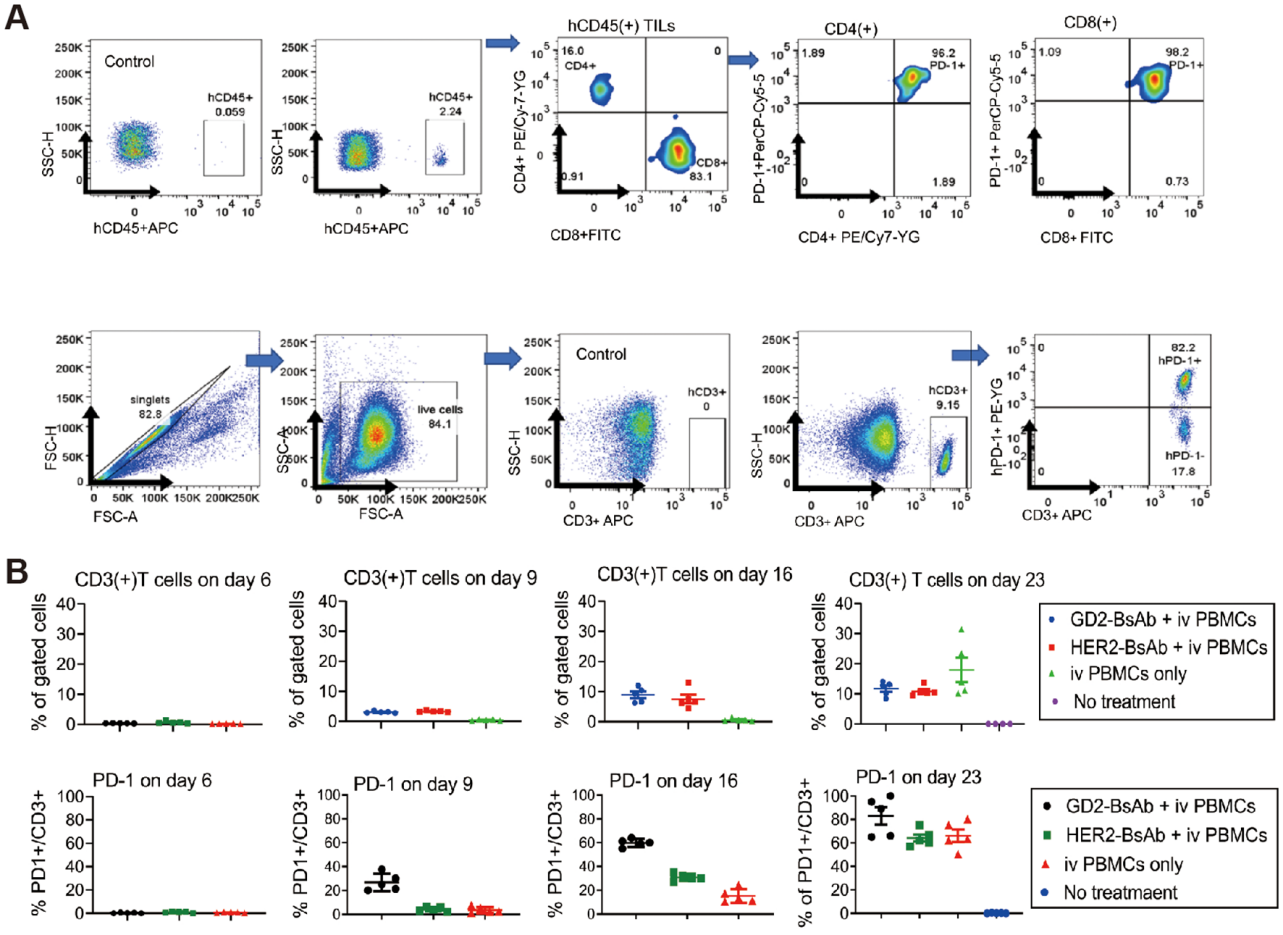
PD-1 and PD-L1 expression by T cells and osteosarcoma cell line xenografts. **(A)** Flow cytometry analysis of PD-1 expression on tumor-infiltrating lymphocytes (TILs) in osteosarcoma 143B cell line xenografts on day 35 post-GD2-BsAb treatment. **(B)** Flow cytometry analyses of human CD3(+) T cells and human PD-1 expression by CD3(+) T cells in peripheral blood after GD2-BsAb or HER2-BsAb treatment. c IHC staining and flow cytometry analysis of human PD-L1 in osteosarcoma 143B xenografts. PD-L1 expression levels were quantified using geometric MFI (MFI) (77).

Therapies targeting specific tumor-specific antigens have been increasing in recent years. Roth et al. performed immunohistochemistry on 44 osteosarcoma specimens and found that GD2 was expressed in all 44 specimens and stained significantly more intensely in tissue from patients with recurrence than in tissue from patients with initial detection, suggesting that GD2 could be a target for immunotherapy in osteosarcoma (75). Dinutuximab, an anti-GD2 antibody, was used to enhance the efficacy of chemotherapy in three patients with Ewing sarcoma. All patients receiving the combination therapy tolerated it well, with complete tumor remission and no signs of recurrence, making GD2 an essential target for bone tumor immunotherapy. Theruvath et al. combined anti-GD2 therapy with anti-CD47 therapy, which upregulated calreticulin expression on the surface of osteosarcoma cells, respectively, as well as promoting phagocytosis of osteosarcoma cells by macrophages (76). Combining the two therapies in MG63 and 143B cell lines effectively inhibited tumor cell proliferation and successfully activated macrophages in the tumor immune microenvironment. Park et al. used anti-GD2 bis-specific antibodies (BsAbs) and anti-HER2-BsAbs to treat osteosarcoma. T cell engaging bispecific antibodies (T-BsAbs) using sequences of anti-CD3 (huOKT3) and anti-disialoganglioside [GD2] (hu3F8) or anti-epidermal growth factor receptor-2 [HER2] (trastuzumab) antibody structured on IgG-[L]-scFv format with silenced Fc, exerting potent anti-tumor activities. The CAR-T cells could effectively recruit T cells, and using them in combination with anti-PD-L1 antibodies to treat osteosarcoma is also a direction to be considered (**Figure 6**) (77).

## 3 Reverse the suppression in the tumor immune microenvironment

Some immune cells and components of the extracellular matrix become immunosuppressive factors in the tumor immune microenvironment as the tumor progresses. They may directly or indirectly contribute to immune escape from the tumor (69, 78). In immunotherapy of bone tumors, reversing the immunosuppressive state caused by these factors is the primary goal of treatment (79–81). In this paper, we review the reverse of immunosuppression by regulatory T cells, tumor-associated macrophages, and bone marrow-derived suppressor cells and by remodeling the tumor’s extracellular matrix, thus reversing the immunosuppressive state of the tumor immune microenvironment.

### 3.1 Reversing immunosuppression against regulatory T cells

Regulatory T cells (Tregs) are a subpopulation of T cells with immunosuppressive functions, usually expressing CD4, CD25, and FOXP3 as surface markers on the cell surface (82). Treg cells, which usually have potent immunosuppressive functions in the tumor immune microenvironment, have become a hot spot for research in recent years (83). IL-35 is the main cytokine secreted by Treg cells, and Liu et al. found that IL-35 levels significantly increased in osteosarcoma patients’ blood. At the same time, IL-35 decreased the anti-tumor activity of CD8+ T cells (84). In an analysis of immune cell infiltration in tumor specimens from osteosarcoma patients by Sun et al., Treg cells exhibited the same immunosuppressive capacity as peripheral blood Treg cells. At the same time, Biller et al. showed that in a canine osteosarcoma model, Treg cell numbers were significantly increased, and the CD8/Treg ratio was significantly correlated with prognosis in dogs (85, 86). A multicenter validation of a retrospective study of patients with osteosarcoma demonstrated increased mortality in patients with increased Treg cell content in osteosarcoma specimens. Brinkrolf et al. also found a similar profile of Treg cells in Ewing sarcoma (87). Therefore, reducing Treg cell infiltration in tumor tissues has become a developmental direction for immunotherapy of bone tumors.

**Figure 7:**
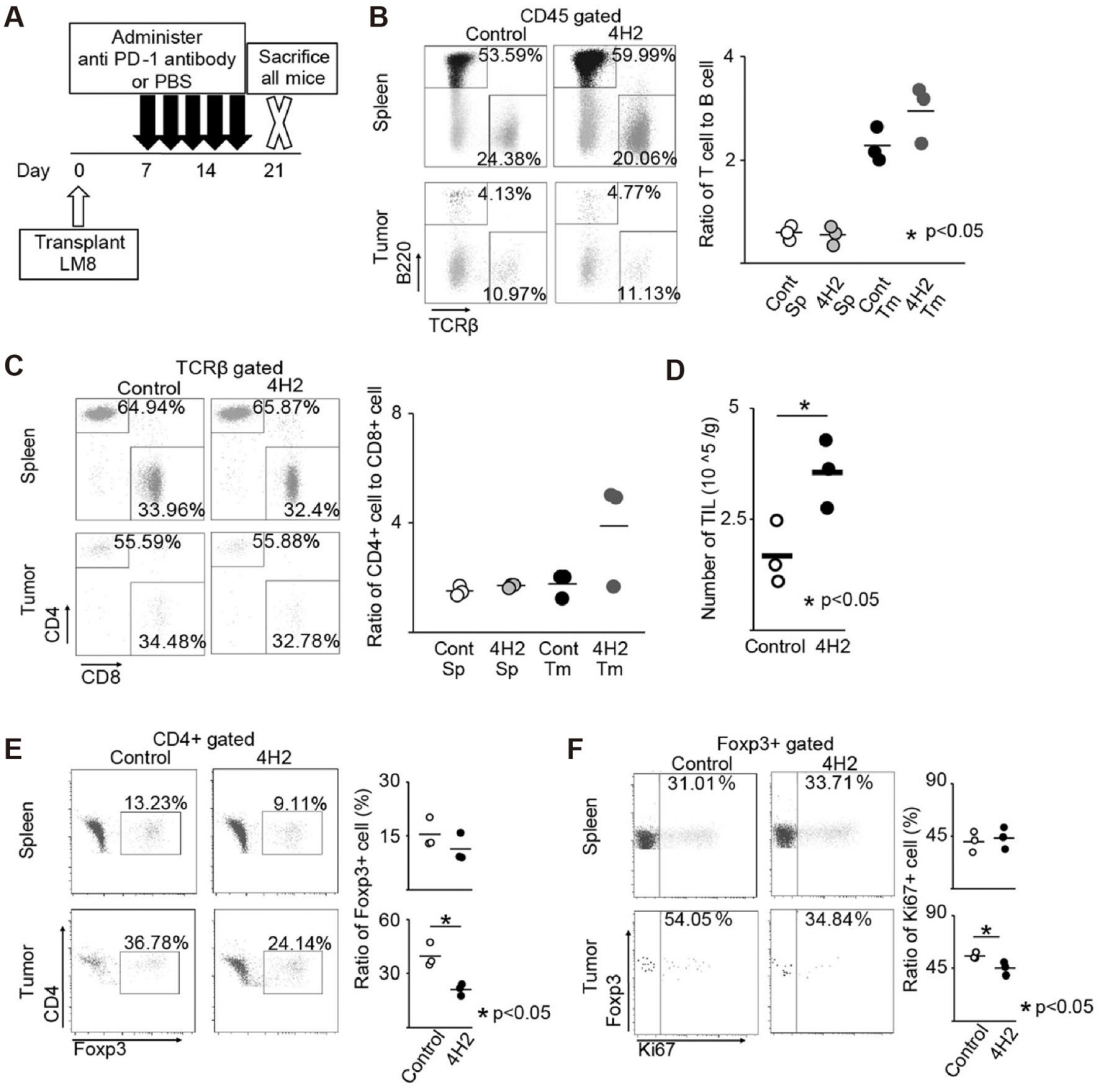
Anti-PD-1 antibody changes the tumor microenvironment. The spleen and tumor immune cell proportion were evaluated (n = 3). The representative specimen is shown left. Each specimen is plotted, and the average value is indicated by a horizontal bar, right. **(A)** Schema of experimental overview. **(B)** Ratio of T cell to B cell. **(C)** Ratio of CD4 + cell to CD8 + cell in TCRβ + cells. **(D)** Number of TILs recovered from the tumor per unit weight. **(E)** Percentage of Foxp3 + cells in CD4 + cells. **(F)** Percentage of Ki-67 + cells in Foxp3 + cells (89).

Mortara et al. treated osteosarcoma with L19TNF-a (L), marfalan (M), and gemcitabine (G) showed a decrease in Treg cells, myeloid suppressor cells (MDSCs), and a significant increase in CD4+ and CD8+ T cells in tumor tissue (88). The group treated with the L-M-G-G regimen demonstrated more potent anti-tumor activity. Since Treg cells express PD-1 and CTLA-4, in recent years, several checkpoint inhibitors have been found to reduce Treg cell infiltration in tumors. Yoshida et al. first reported that anti-PD-1 antibodies reduced Treg infiltration in a mouse model of osteosarcoma (**Figure 7**) (89). In bone and synovial sarcoma cell lines, Treg is usually induced to mature by DC cells. In the study by Ocadlikova et al., osteosarcoma and synovial sarcoma cells were treated with the PD-1 inhibitor sunitinib, and the ability of DC to induce Treg cell maturation was analyzed after co-culturing them with DC for a while (90). The results showed that the PD-1 inhibitor could completely block the DC-induced maturation of Treg. Takahashi et al. then investigated the efficacy of anti-PD-L1 combined with anti-CTLA-4 antibody P1C4 and radiotherapy in treating osteosarcoma in mice (91). In the combination treatment group, CD8+ T cell infiltration was increased, Treg cell infiltration was decreased, and the CD8/Treg ratio was significantly increased.

### 3.2 Reversing immunosuppression against tumor-associated macrophages

As mentioned previously, tumor-associated macrophages (TAMs) consist of a large number of M2-type macrophages that are immunosuppressive components of the tumor immune microenvironment (92). Numerous studies have shown that M2-type macrophages strongly suppress infiltrating T cells within bone tumors and are an essential target for bone tumor therapy. Han et al. reported that removing M2-type (CD163+), macrophages increased the number of T cells in tumor tissue from patients with osteosarcoma (93). In addition, M2-type macrophage cells can repolarize to M1-type macrophages, which has attracted the attention of researchers, and modulation of TAM repolarization to M1-type macrophages has become one of the current means of immunotherapy for bone tumors (94).

**Figure 8:**
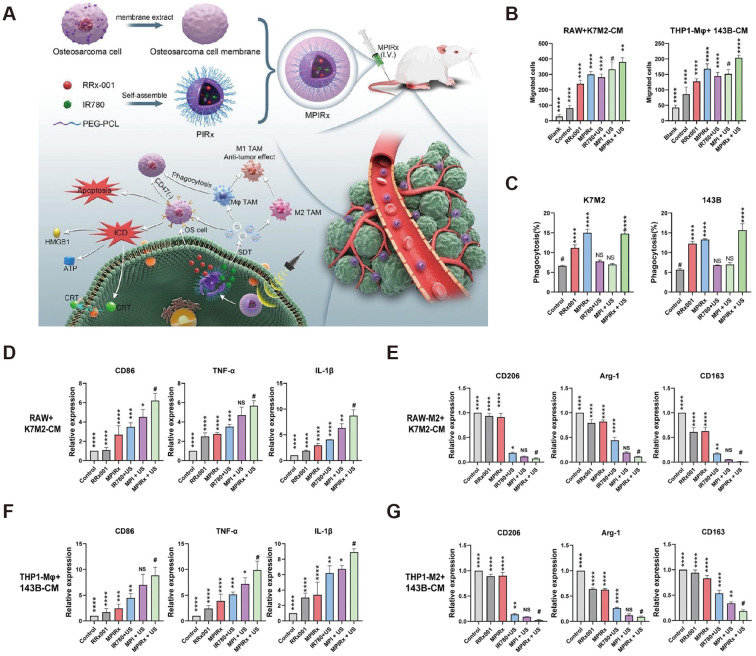
Regulation of macrophages by MPIRx nanodrugs. **(A)** Construction of MPIRx nanodrugs for CD47 immune checkpoint/sonodynamic therapy of osteosarcoma and pulmonary metastasis. **(B)** Transwell migration assay of macrophages (THP1-Mφ, RAW264.7) cocultured with OS cells (K7M2, 143B). **(C)** Flow cytometry analysis of phagocytosis. **(D, F)** qPCR analysis of M1 TAMs related markers in macrophages treated with conditioned medium of OS cells. **(E, G)** qPCR analysis of M2 TAMs related markers in M2-macrophages treated with OS-CM (100).

Zhou et al. found a significant reduction in pulmonary metastases when treating osteosarcoma with all-trans retinoic acid (95). Further study found that all-trans retinoic acid inhibited TAM polarization to M2-type and thus inhibited tumor cell metastasis. Shao et al. further found that all-trans retinoic acid inhibited osteosarcoma cells’ proliferation and differentiation ability by promoting TAM repolarization and decreasing the expression of tumor stem cell surface markers (96). Fujiwara et al. found that CSF1R inhibitors modulated TAM (97). The CSF1R inhibitor Pexidartinib prevented TAM from being stimulated to differentiate into M2-type macrophages by CSF1 secreted by tumor cells. In the mouse osteosarcoma model, treatment with Pexidartinib significantly reduced TAM and Treg cells and increased the infiltration of CD8+ T cells. Li et al. regulated TAM repolarization by inhibiting the macrophage PI3K pathway and blocking CSF1R (98). They used nano micelles loaded with PI3K inhibitor BEZ 235 and CSF1R-siRNA to reduce M2-type macrophage levels in tumor tissues through the synergistic effect of both. Gong et al. designed a nanomedicine that combines CD47 inhibition therapy and acoustic kinetic therapy (99). The nanomedicine was loaded with the CD47 inhibitor RRx-001 and the acoustic sensitizer IR780, and the two drugs were delivered to the tumor site to inhibit the proliferation and migration of osteosarcoma cells, reduce the expression of the “don’t-eat-me signal” on the surface of the tumor cells, and regulate the polarization of macrophages toward the anti-tumor M1 phenotype (**Figure 8**). Reximod (R848), a common Toll-like receptor inhibitor, has also been used to modulate M2-type macrophage repolarization. In one study, adriamycin, cisplatin, and R848 were loaded in nanoparticles with sustained drug release (100). After reaching the tumor site by intravenous injection, adriamycin, and cisplatin triggered immunogenic cell death to kill tumor cells, while R848 promoted repolarization of M2-type macrophages and enhanced phagocytosis of TAM, acting synergistically for the treatment of osteosarcoma.

### 3.3 Reversing immunosuppression against myeloid-derived suppressor cells

Myeloid-derived suppressor cells (MDSCs) are a heterogeneous population of cells with immunosuppressive properties, mainly immature granulocytes, DCs, and macrophages (101). MDSCs are more abundant in the bone tumor microenvironment than in other solid tumors. MDSC can directly suppress the function of CD4+ T cells, CD8+ T cells, DCs, and NK cells and also promote the production of Treg cells (102). Researchers have used various methods to reduce the number of MDSCs and decrease the immunosuppressive effects.

**Figure 9:**
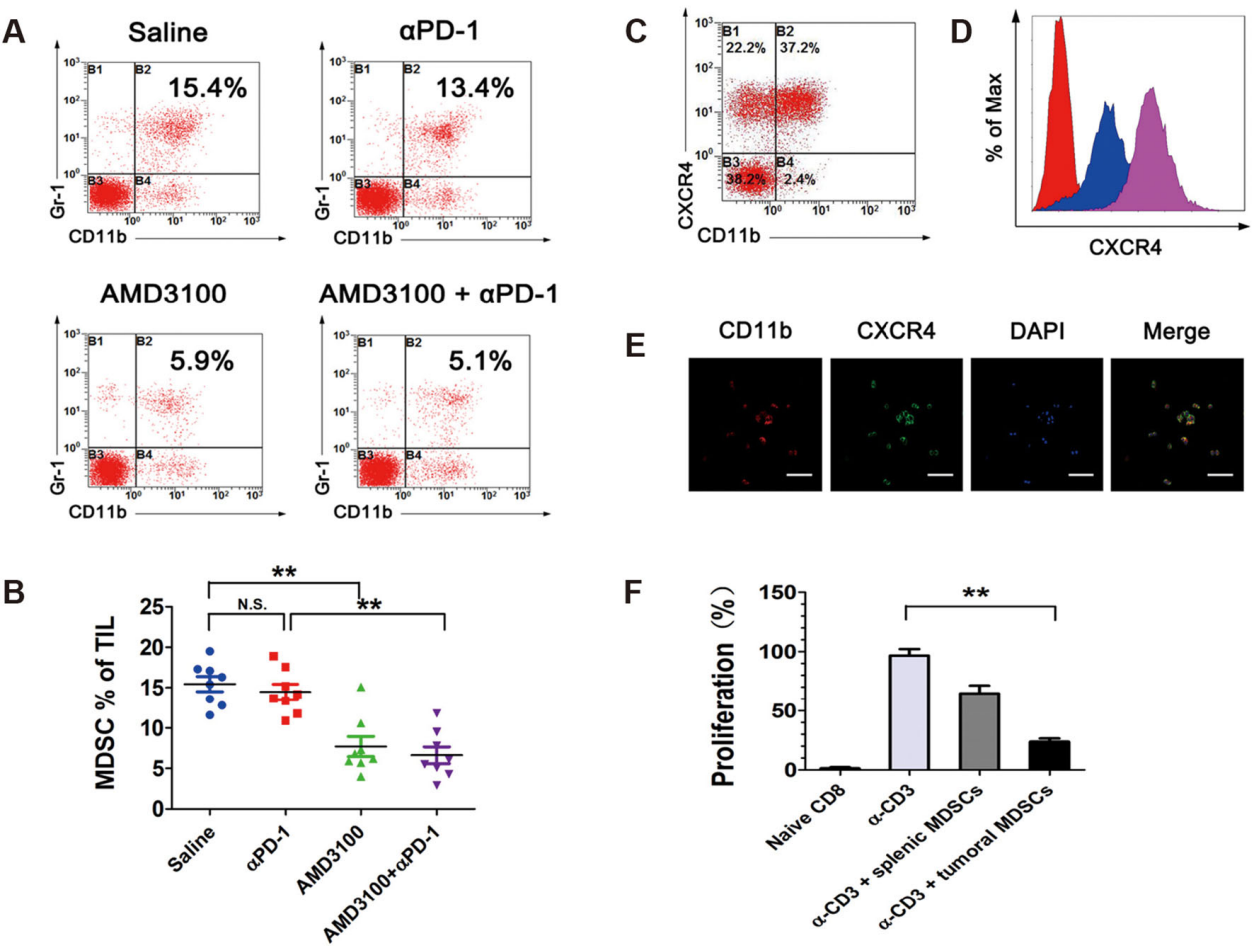
Tumor-infiltrating MDSCs express high levels of CXCR4 and are diminished by AMD3100 administration in osteosarcoma. **(A, B)** Percentages of Gr-1+/CD11b+ MDSCs in intratumoral CD45+ cell population in different treatment groups, measured by flow cytometry. **(C)** Percentages of CXCR4+ MDSCs in intratumoral Gr-1+/CD11b+ cell population. **(D)** Expression levels of CXCR4 in splenic MDSCs (blue) and intratumoral MDSCs (purple), measured by flow cytometry. **(E)** Immunofluorescence microscopy analysis of CD11b and CXCR4 in FACS-sorted MDSCs. **(F)** Splenic MDSCs and intratumoral MDSCs were placed in a proliferation assay with CFSE-labeled CD8+ T cells at the designated MDSC/T cell ratios. Anti-CD3/CD28 beads were used at a 1:2 ratio with T cells to induce proliferation. Cells were harvested on day 5 and analyzed by FACS for Violet dilution (104). * *P*< 0.05, ** *P*< 0.01, *** *P*< 0.001.

Horlad et al. found that Corosolic acid inhibits the immunosuppressive activity of MDSCs in 2013 (103). Jiang et al. found that the SDF-1/CXCR4 axis promotes MDSC aggregation in the osteosarcoma microenvironment (**Figure 9**) (104). They used the CXCR4 antagonist AMD3100 to block the binding of SDF-1 to CXCR4, which significantly reduced the infiltration of MDSCs in osteosarcoma and had a synergistic effect when combined with anti-PD-1 antibodies, a novel option for immunotherapy of osteosarcoma. In a study by Shi et al., the PI3Kδ/γ inhibitor (S)-(-)-N-[2-(3-hydroxy-1H-indol-3-yl)-methyl]-acetamide (SNA) enhanced the efficacy of anti-PD-1 antibodies in the treatment of osteosarcoma, and further studies showed that SNA inhibited MDSCs thereby enhancing the anti-tumor effects of CD8+ T cells (105). Long et al. used all of The abilities of trans-retinoic acid to inhibit MDSCs from enhancing the efficacy of CAR-T cell therapy (106). In addition, metformin was found to inhibit both MDSCs and TAM immunosuppression in the osteosarcoma microenvironment.

### 3.4 Reverse the immune suppression in tumor extracellular matrix

In addition to cells that cause immunosuppression in the tumor immune microenvironment, many tumor-derived immunosuppressive factors, including cytokines, small molecules, and immunosuppressive enzymes, are also present in the tumor extracellular stroma (69). These immunosuppressive factors accumulate in the tumor microenvironment and inhibit the immune cells that enter it. In response to these immunosuppressive factors, attempts have been made to relieve the immunosuppressive state of tumors by remodeling the extracellular matrix and reducing the secretion of immunosuppressive factors (107, 108). Here we focus on using various immunosuppressive enzymes as immunotherapeutic targets for treating bone tumors.

COX-2 was discovered in 1991 and has long been thought to be involved in cancer progression. In normal cells, COX-2 levels are low. However, in tumor tissues, they are significantly elevated. COX-2 levels in osteosarcoma are strongly associated with disease progression and prognosis. Zhao et al. demonstrated how COX-2 promotes osteosarcoma progression (109). They investigated the effect of COX-2 on the proliferation, migration, and other properties of osteosarcoma cells using a SaOS2 human osteosarcoma cell line with the COX-2 gene knocked out. Chen et al. demonstrated the inhibitory effect of euxanthone on COX-2 in osteosarcoma cell lines, but euxanthone could not wholly block the expression of COX-2. Hence, the investigators speculated that osteosarcoma should have other pathways that also lead to COX-2 expression, and how to completely block COX-2 expression in bone tumor cells becomes an issue that needs to be addressed now (110). Similarly, indoleamine 2,3-dioxygenase (IDO) plays an immunosuppressive role in various tumor microenvironments, and Liebau et al. showed as early as 2002 that IFN-γ, IL-12, and IL-18 all induced IDO expression in human osteosarcoma cell lines (111). MAX et al., on the other hand, used transgenic techniques to study the CD137/CD137L pathway on Ewing sarcoma expression of IDO regulation of the CD137L transgenic tumor cells expressing IDO was significantly suppressed, and regulation of CD137/CD137L pathway was effective in reducing the expression of Ewing sarcoma cells by IL-2 stimulated expression of IDO (112). Inducible nitric oxide synthase (iNOS) is also of interest for its immunosuppressive ability in the tumor microenvironment, and its expression in humans often indicates iNOS has now been shown to be highly expressed in a variety of solid tumors, in general, iNOS expression is closely associated with tumor progression. iNOS was shown to promote osteosarcoma development via the Wnt/β-linked protein pathway in iNOS knockout mice, and tumor progression was significantly inhibited by Chu et al. (113). In bone tumor immunotherapy, several of the above enzymes should be given adequate attention, and the immunosuppressive state of the tumor immune microenvironment can be effectively relieved by blocking the expression of one or more immunosuppressive enzymes.

## 4 Discussion and Conclusions

Osteosarcoma, as a highly malignant tumor with a distinct and suppressive immune microenvironment, presents unique challenges for effective immunotherapy. While advancements have been made in modulating immune responses and targeting the tumor immune microenvironment, the outcomes remain suboptimal compared to other solid and hematological malignancies. Current immunotherapy for osteosarcoma has several shortcomings, including the response rate of osteosarcoma patients to immunotherapy, large individual differences in treatment efficacy, the immunosuppressive microenvironment that affects the efficacy of immunotherapy, and the lack of molecular targets specific to osteosarcoma. These issues represent challenges and opportunities, and when they are resolved, immunotherapy for osteosarcoma is expected to completely replace conventional therapies and prolong the survival of patients with distant metastases.

Immunotherapy for osteosarcoma currently faces many challenges in clinical application. The first step is to be fully aware of the complexity of the immune microenvironment. An in-depth understanding of the immunosuppressive network in osteosarcoma is crucial. High-throughput technologies such as single-cell RNA sequencing and spatial transcriptomics can elucidate cell-cell interactions and identify new therapeutic targets. Secondly, TME is characterized by resistance mechanisms such as the recruitment of regulatory T cells (Tregs), tumor-associated macrophages (TAMs), and myeloid-derived suppressor cells (MDSCs). Reversing these inhibitory effects will require the development of combination therapies and biomaterials with controlled delivery. At the same time, given the heterogeneity of osteosarcoma, personalized treatment regimens tailored to a patient’s molecular and immunological profile are likely to improve outcomes. Such an approach would require integrated analyses of genomic, proteomic, and immunological data. The combination of immunotherapy with other treatments is one of the current research breakthroughs needed.The current difficulties are mainly centered on the fact that the mechanism of combining immunotherapy with other therapeutic approaches is still unclear, which challenges the optimal dosage, timing and safety of these combinations.

In response to the current status of immunotherapy for osteosarcoma, we believe that future research should enhance targeting through combination therapies, harness the power of biomaterials to exert synergistic effects with conventional therapies, and reverse the immune evasion mechanism of tumors. Firstly in preclinical models, combining immune checkpoint inhibitors (ICIs) such as anti-PD-1/PD-L1 antibodies with drugs that can restore polarity to TAMs or enhance T cell function has shown promise. CAR-T cell therapies targeting antigens such as HER2 and B7-H3 in combination with immune checkpoint blockers can extend the therapeutic effect. Secondly leveraging nanotechnology innovations such as nanoparticle-mediated delivery of cytokines or small molecule inhibitors can optimize pharmacokinetics, reduce systemic toxicity, and ensure more precise modulation of TME, while immunotherapies effectively synergize with chemotherapy and radiotherapy by enhancing tumor antigen presentation and immune cell infiltration. Optimizing the timing and dosage of these combination therapies will maximize treatment efficacy. In addition, future studies should focus on targeting extracellular matrix remodeling and immunosuppressive enzymes such as COX-2 and IDO, which play a key role in immune evasion.

The emergence of lysogenic viruses has provided new ideas for immunotherapy of osteosarcoma. Adenoviruses are genetically engineered to remove genes such as E1A or E1B, allowing them to selectively infect and lyse osteosarcoma cells and activate the immune system to clear the tumor. Herpes simplex virus has also been used to treat osteosarcoma. Herpes simplex virus with the ICP34.5 gene removed can effectively inhibit the growth of osteosarcoma, and at the same time, combined with radiotherapy or chemotherapy, the efficacy is more obvious. With the approval of oncolytic viruses for other solid tumors, the use of oncolytic viruses for the treatment of osteosarcoma has also become possible.

The field of osteosarcoma immunotherapy is poised at a critical juncture, where novel insights into the tumor immune microenvironment and advancements in immunomodulatory strategies are paving the way for more effective treatments. Despite significant progress, challenges persist in translating preclinical findings into clinical success. To achieve meaningful therapeutic outcomes, a paradigm shift toward multi-faceted and personalized therapeutic regimens is imperative. This includes leveraging advanced biomaterials, designing innovative combination therapies, and integrating omics data to tailor treatments to individual patients. Furthermore, establishing robust clinical trial frameworks to evaluate these strategies is essential for their successful implementation. Ultimately, the goal is to develop immunotherapy approaches that not only complement existing modalities but also provide standalone therapeutic options that can replace traditional cytotoxic treatments. The collective efforts of researchers, clinicians, and policymakers will be vital in overcoming these challenges and advancing immunotherapy to the forefront of osteosarcoma treatment.
